# Enhanced efficacy of histone deacetylase inhibitor combined with bromodomain inhibitor in glioblastoma

**DOI:** 10.1186/s13046-018-0916-y

**Published:** 2018-10-01

**Authors:** Wei Meng, Baocheng Wang, Weiwei Mao, Jiajia Wang, Yang Zhao, Qifeng Li, Chenran Zhang, Yujie Tang, Jie Ma

**Affiliations:** 1 Department of Pediatric Neurosurgery, Xin Hua Hospital Affiliated with Shanghai Jiao Tong University School of Medicine, Shanghai, 200092 People’s Republic of China; 2 Key Laboratory of Cell Differentiation and Apoptosis of the National Ministry of Education, Department of Pathophysiology, Shanghai Jiao Tong University School of Medicine, 280 South Chongqing Road, Shanghai, 200025 People’s Republic of China

**Keywords:** Glioblastoma, Panobinostat, JQ1, OTX015

## Abstract

**Background:**

Glioblastoma (GBM) is the most common and most malignant primary brain cancer in adults. Despite multimodality treatment, the prognosis is still poor. Therefore, further work is urgently required to discover novel therapeutic strategies for GBM treatment.

**Methods:**

The synergistic effects of cotreatment with the histone deacetylase (HDAC) inhibitor panobinostat and bromodomain inhibitor JQ1 or OTX015 were validated using cell viability assays in GBM cell lines. Furthermore, the inhibitory mechanisms were investigated via an EdU proliferation assay, an apoptosis assay, qPCR, Western blot and RNAseq analyses.

**Results:**

We found that the cotreatment with panobinostat and JQ1 or OTX015 synergistically inhibited cell viability in GBM cells. The cotreatment with panobinostat and JQ1 or OTX015 markedly inhibited cell proliferation and induced apoptosis in GBM cells. Compared with treatment with each drug alone, the cotreatment with panobinostat and JQ1 induced more profound caspase 3/7 activation and cytotoxicity. Mechanistic investigation showed that combination of panobinostat with JQ1 or OTX015 results in stronger repression of GBM-associated oncogenic genes or pathways as well as higher induction of GBM-associated tumor-suppressive genes.

**Conclusion:**

Our study demonstrated that HDAC inhibitor and bromodomain inhibitor had synergistical efficacy against GBM cells. The cotreatment with HDAC inhibitor and bromodomain inhibitor warrants further attention in GBM therapy.

## Background

Glioblastoma multiforme (GBM) is the most common and most malignant primary brain cancer in adults [1]. Despite optimal multimodality treatment consisting of surgical debulking, radiotherapy and temozolomide chemotherapy, the median survival is still 12–15 months [2]. Based on successful preclinical studies, many clinical trials have tested the efficacy of novel therapies, but improvement in the survival of patients with GBM has been limited over the past few decades [3]. Therefore, further work is urgently required to discover novel therapeutic strategies for GBM treatment.

Epigenetic mechanisms are increasingly considered major factors contributing to the pathogenesis of cancer, including glioblastoma [4]. Histone deacetylases (HDACs) are overexpressed and mutated in various solid and hematologic malignancies and play key roles in tumorigenesis [5]. Various HDAC inhibitors, such as panobinostat, vorinostat and valproate, have shown potent efficacy against GBM in preclinical studies, and multiple anti-GBM mechanisms, including the induction of cell cycle arrest, differentiation, apoptosis, autophagic cell death, generation of reactive oxygen species, inhibition of angiogenesis and DNA damage repair (DDR), have been suggested [6–8]. While the results of preclinical studies are encouraging, early clinical trials have only showed a modest benefit [9–12]. Therefore, it is important to explore drug combination strategies to improve efficacy.

Bromodomain proteins, such as BRD3 and BRD4, bind acetylated lysine residues on histone proteins as chromatin readers and play essential roles in the transcription of oncogenes, such as C-MYC, MYCN, BCL2, and FOSL1 [13]. Small-molecule bromodomain inhibitors, such as JQ1 and OTX015, competitively bind acetyl–lysine recognition pockets, displace bromodomain proteins from chromatin, and reduce the expression of oncogenes, leading to cancer cell growth inhibition and apoptosis. Bromodomain inhibitors have shown promising anticancer effects against GBM in vitro and in vivo [13–15]. Recently, bromodomain inhibitors have been shown to have synergistic effects with panobinostat in acute myelogenous leukemia cells [16] and neuroblastoma cells [17]. However, whether panobinostat also has synergistic effects with JQ1 or OTX015 in GBM remains elusive. In this study, we demonstrate that cotreatment with the HDAC inhibitor panobinostat and the bromodomain inhibitor JQ1 or OTX015 has synergistic efficacy against GBM in vitro. Cotreatment with the HDAC inhibitor and bromodomain inhibitor warrants further attention in GBM therapy.

## Methods

### Compounds and cell lines

Panobinostat (S1030), JQ1 (S7110) and OTX015 (S7360) were purchased from Selleck Chem (Houston, TX, USA). Human cells used were approved by patients and ethnics committee of Ren Ji Hospital affiliated to Shanghai Jiao Tong University School of Medicine. The U87 and U251 cell lines were obtained from the Cell Bank of the Chinese Academy of Science (Shanghai, China). GBM06 primary cell lines were established from tumor tissues of patients from the Department of Neurosurgery of Ren Ji Hospital. Briefly, Tumors were dissociated into single cells by placing in TrypLE™ Express Enzyme (Life technologies, 12604–021) for 15 min at 37 °C. Dissociated cells were initially allowed to form spheres/aggregates in suspension culture, and then transferred to a fresh flask coated with laminin (Sigma, L2020). U87 and U251 were cultured in Dulbecco’s modified Eagle medium/High glucose (HyClone, Logan, Utah, USA) supplemented with 10% fetal bovine serum, penicillin (100 U/mL) and streptomycin (100 mg/mL). GBM06 were cultured using NeuroCult NS-A Proliferation Kit (Human) (Stem Cell Technology, 05751) supplemented with human EGF-basic (20 ng/ml) (PeproTech, AF-100-15-100), human FGF-basic (20 ng/ml) (PeproTech, 100-18B-100), and 0.2% Heparin Solution (10 ng/ml) (Stem Cell Technology, 07980).

### Cell viability assays

For the cell viability measurements, the cells were plated in 96-well plates in at least triplicate and then subjected to drug treatment as indicated. Then, the cell viability was measured by using a Celltiter Glo assay (G7571, Promega, WI, USA). The data were collected using a Synergy H4 Hybrid Reader (BioTek, Winooski, VT, USA).

### RNA extraction and RT-qPCR

RNA was isolated from the cell lines by TRIzol reagent (Thermo Fisher Scientific) and measured using a Nanodrop 2000 spectrophotometer. Equal amounts of RNA were converted to cDNA using a High Capacity cDNA Reverse Transcription Kit (4368813, Thermo scientific). RT-qPCR was performed in 384-well plates using a 7900 HT fast real-time PCR system (Thermo Fisher Scientific). The fold change in gene expression was calculated using the ddCt method and GAPDH as a reference gene. The following qPCR primer sequences were used:

- CCND1-F TTCAAATGTGTGCAGAAGGA
- CCND1-R GGGATGGTCTCCTTCATCTT
- MKI67-F TTGGAGAATGACTCGTGAGC
- MKI67-R CGAAGCTTTCAATGACAGGA
- TOP2A-F ACTGAAGGAAGCCCTCAAGA
- TOP2A-R TGTTTTTGTTGCTGCTCTCC
- BIRC5-F AGCCCTTTCTCAAGGACCAC
- BIRC5-R CAGCTCCTTGAAGCAGAAGAA
- BCL-XL-F CTGAATCGGAGATGGAGACC
- BCL-XL-R TGGGATGTCAGGTCACTGAA
- MYC-F AGAGTCTGGATCACCTTCTGCT
- MYC-R ACACTGTCCAACTTGACCCTCT
- BCL2-F: GAGAAATCAAACAGAGGCCG
- BCL2-R: CTGAGTACCTGAACCGGCA
- BCL3-F: CCGGAGGCGCTTTACTACC
- BCL3-R: TAGGGGTGTAGGCAGGTTCAC
- VEGFC-F: GAGGAGCAGTTACGGTCTGTG
- VEGFC-R: TCCTTTCCTTAGCTGACACTTGT
- WEE1-F: AGGGAATTTGATGTGCGACAG
- WEE1-R: CTTCAAGCTCATAATCACTGGCT
- PBK-F: CCAAACATTGTTGGTTATCGTGC
- PBK-R: GGCTGGCTTTATATCGTTCTTCT
- CDC20-F: GCACAGTTCGCGTTCGAGA
- CDC20-R: CTGGATTTGCCAGGAGTTCGG
- FOXO3-F: CGGACAAACGGCTCACTCT
- FOXO3-R: GGACCCGCATGAATCGACTAT
- P21-F: TGTCCGTCAGAACCCATGC
- P21-R: AAAGTCGAAGTTCCATCGCTC
- BNIP3-F: CAGGGCTCCTGGGTAGAACT
- BNIP3-R: CTACTCCGTCCAGACTCATGC
- SOD2-F: GCTCCGGTTTTGGGGTATCTG
- SOD2-R: GCGTTGATGTGAGGTTCCAG
- GADD45A-F: GAGAGCAGAAGACCGAAAGGA
- GADD45A-R: CAGTGATCGTGCGCTGACT
- GADD45B-F: TACGAGTCGGCCAAGTTGATG
- GADD45B-R: GGATGAGCGTGAAGTGGATTT

### Cell proliferation and apoptosis assays

Cell proliferation was measured by using a Click-iT EdU Alexa Fluor 647 Flow Cytometry Assay Kit (C10640, Invitrogen, CA, USA). EdU+ population represents the proliferating cell population. Cell apoptosis was measured by using an Annexin V-FITC Apoptosis Detection Kit I (556547, BD Biosciences, CA, USA) with some minor modifications. DAPI was used instead of PI. The FACS analyses were performed by using a BD Fortessa FACS machine (BD Biosciences, CA, USA). The data were analyzed using FlowJo software (FlowJo, LLC, OR, USA).

### Caspase and cytotoxicity assays

For the caspase and cytotoxicity measurements, the cells were plated in 96-well plates in at least triplicates and then subjected to drug treatments as indicated. Then, the cell caspase activity and cytotoxicity were measured by using a Caspase 3/7 Glo assay (G8092, Promega, WI, USA) and CytoTox-Glo™ Cytotoxicity Assay (G9292, Promega, WI, USA), respectively, and the data were collected using a Synergy H4 Hybrid Reader (BioTek, Winooski, VT, USA).

#### Western blotting

Cells were lysed in radioimmunoprecipitation assay (RIPA) buffer with 1X protease inhibitor cocktail (Roche 0469313200) and 1% phosphatase inhibitor cocktail (Sigma P0044). Pierce™ Bicinchoninic Acid (BCA) Protein Assay Kit (Thermo Fisher Scientific) was used to calculate protein concentration according to the manufacturer’s protocol. Sodium dodecyl sulfate polyacrylamide gel electrophoresis (SDS-PAGE) was performed and proteins were transferred to Polyvinylidene difluoride (PVDF) Membrane (Millipore). Membranes were incubated for 16–20 h at 4 °C with primary antibodies: Akt (pan) (1,1,000, Cell Signaling Technology #2920), Phospho-Akt (Ser473) (1,1,000, Cell Signaling Technology #4060), c-Myc (1,1,000, Cell Signaling Technology #5605S) and Beta-Tubulin (1,5000, Abcam #ab6046).

### RNA sequencing

The RNA sequencing was performed by OE Biotech (Shanghai, China). Briefly, U87 cells treated with DMSO, panobinostat (0.05 μM), JQ1 (1 μM) or panobinostat/JQ1 cotreatment for 16 h were collected in biological duplicates. The cells were lysed in TRIzol reagent and frozen at − 80 °C. The total RNA was extracted using a mirVana miRNA Isolation Kit (Ambion) following the manufacturer’s protocol. The RNA integrity was evaluated using an Agilent 2100 Bioanalyzer (Agilent Technologies, Santa Clara, CA, USA). Samples with an RNA Integrity Number (RIN) ≥ 7 were included in the subsequent analysis. The libraries were constructed using a TruSeq Stranded mRNA LTSample Prep Kit (Illumina, San Diego, CA, USA) according to the manufacturer’s instructions. Then, these libraries were sequenced on an Illumina sequencing platform (HiSeqTM 2500 or Illumina HiSeq X Ten), and 125 bp/150 bp paired-end reads were generated.

### RNAseq data processing

First, we performed adaptor trimming with cutadapt using the paired-end sequencing data generated in this manuscript (https://cutadapt.readthedocs.io/en/stable/index.html). Then, STAR (version 020201) was used to align the paired-end reads to the reference genome hg19 [18]. The FPKM values of each gene were calculated by cufflinks (version 2.2.1) [19, 20]. The total read counts of each gene were calculated by FeatureCounts [21]. We defined the active genes as genes with FPKM values above 1. The significantly differentially expressed genes (FDR ≤ 0.05) were identified by DESeq [22]. A Gene Oncology (GO) enrichment analysis of the differentially expressed genes was performed by using a hypermetric distribution to compute *P*-values with KEGG pathways and C5-biological process gene sets from the Molecular Signature Database.

### Statistical analyses

Two-tailed Student’s t-test was used to compare two groups. *p < 0.05, ** *p* < 0.01, *** *p* < 0.001.

## Results

### Cotreatment with an HDAC inhibitor and a bromodomain inhibitor synergistically suppresses cell growth in GBM cells

**Fig. 1:**
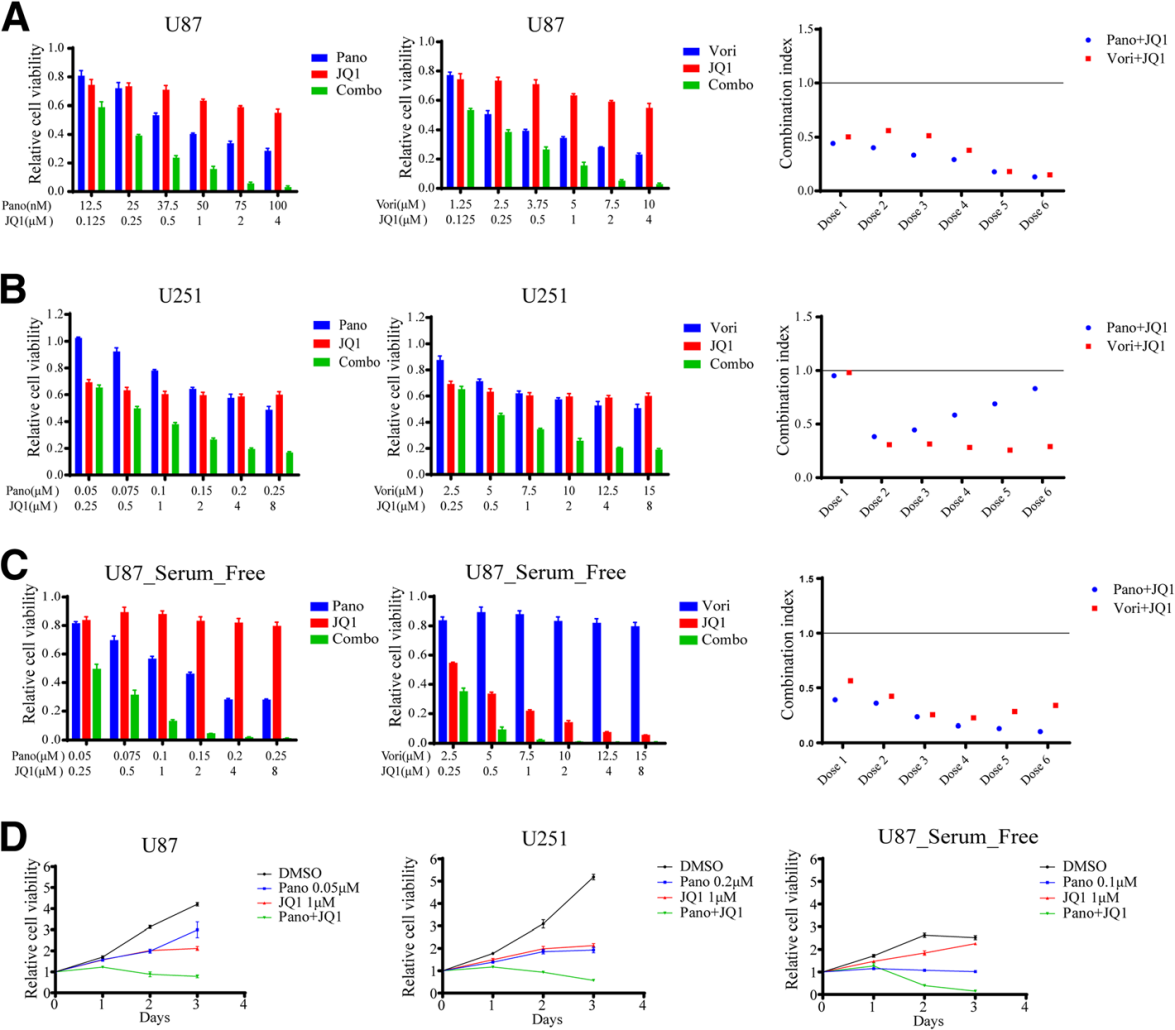
Cotreatment with a HDAC inhibitor and bromodomain inhibitor synergistically inhibits cell viability in GBM cells. **a**-**c** Cotreatment with a HDAC inhibitor (panobinostat or vorinostat) and bromodomain inhibitor JQ1 at various indicated dosages synergistically inhibits cell viability in U87, U251 and serum-free cultured U87 cells. The combination indices are shown on the left. **d** Time course tracking of cell viability after the cotreatment with panobinostat and JQ1 at various indicated dosages in U87, U251 and serum-free cultured U87 cells

First, we tested the combination of an HDAC inhibitor and a bromodomain inhibitor to explore the possible synergistic inhibition effect on GBM cells. The combination index (CI) was used to determine whether the combined treatment of the drugs is synergistic, additive, or antagonistic by the Chou and Talalay method [23]. GBM cell lines, including U87, U251 and serum-free cultured U87 (to mimic the stem cell culture condition) cells, were exposed to increasing concentrations of an HDAC inhibitor (panobinostat or vorinostat) or the bromodomain inhibitor JQ1 for 72 h. As shown in Fig. 1a-c, the combined treatment with the HDAC inhibitor (panobinostat or vorinostat) and bromodomain inhibitor JQ1 resulted in a sharp dose-dependent decline in cell viability in each cell line tested. In contrast, the individual administration of these agents only had minimal effects. As shown in Fig. 1a-c, instead of a simple additive killing effect, the HDAC inhibitor and bromodomain inhibitor combination exerted a highly synergistic inhibition effect on GBM cells with a CI value well below 1. Then, we treated the U87, U251 and serum-free cultured U87 cell lines with DMSO, panobinostat, JQ1 or panobinostat/JQ1 for 24 h, 48 h or 72 h, and the results showed that the panobinostat and JQ1 treatment caused a time-dependent growth disruption in GBM cells (Fig. 1d). Compared with the treatment with each agent alone, the combined treatment of the GBM cells with panobinostat and JQ1 induced greater inhibition of cell growth in a time-dependent manner. These results indicate that the cotreatment with panobinostat and JQ1 synergistically inhibits cell viability in a dose- and time-dependent manner in GBM cells.

### Cotreatment with panobinostat and JQ1 markedly inhibits cell proliferation and induces apoptosis in GBM cells

**Fig. 2:**
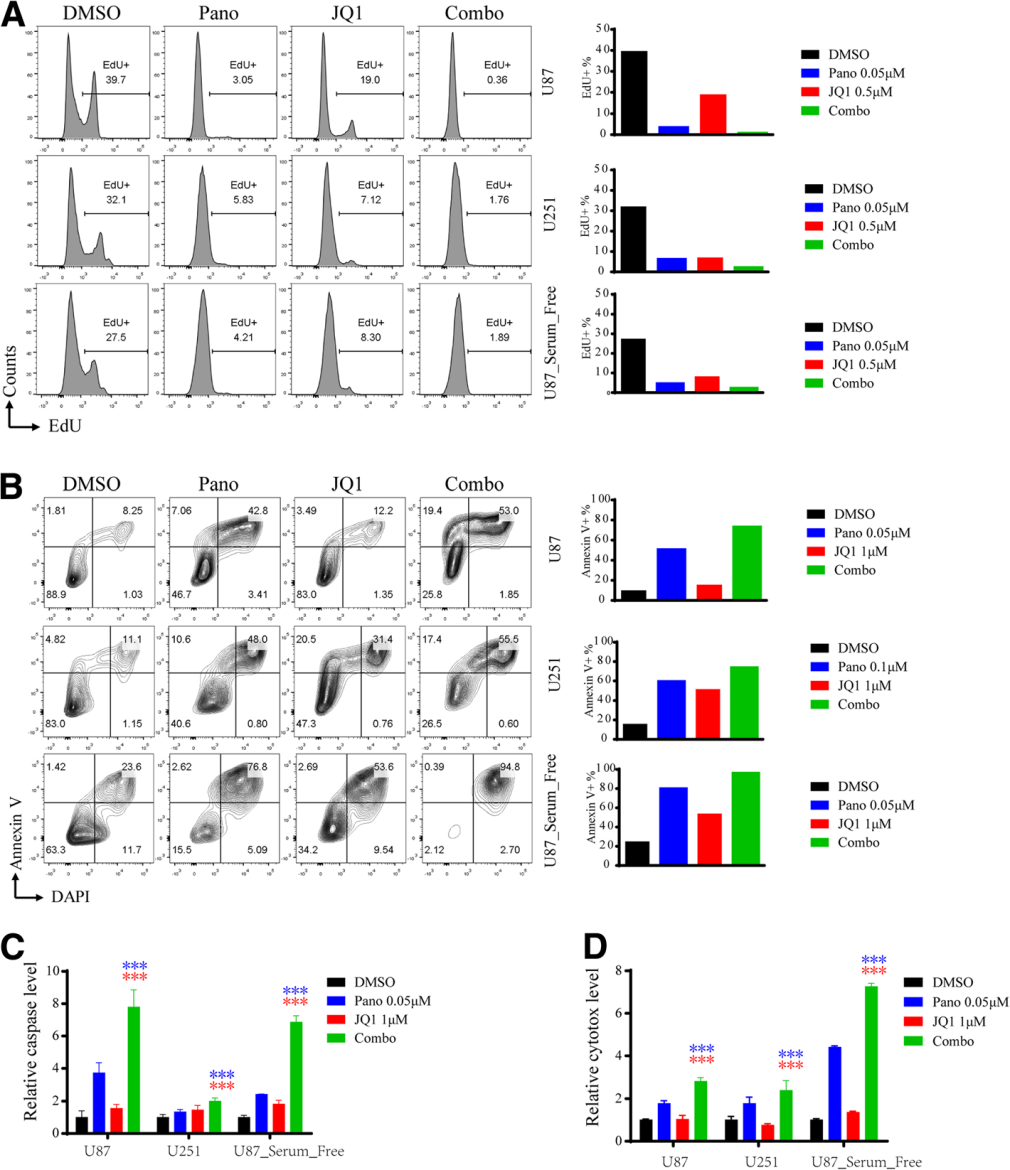
Cotreatment with panobinostat and JQ1 markedly inhibits cell proliferation and induces apoptosis in GBM cells. **a** Cell proliferation analyses of GBM cells treated with DMSO, panobinostat, JQ1 or panobinostat/JQ1 for 24 h using an EdU incorporation FACS assay. Percentages of EdU+ cells are presented on the bar chart on the right. **b** Apoptosis analyses of GBM cells treated with DMSO, panobinostat, JQ1 or panobinostat/JQ1 for 48 h by an Annexin-V staining FACS assay. Percentages of Annexin-V+ cells are presented on the bar chart on the right. **c** Caspase 3/7 activity in GBM cells treated with DMSO, panobinostat, JQ1 or panobinostat/JQ1 at the indicated concentrations for 48 h using a Caspase-Glo assay. **d** Cytotoxicity of GBM cells treated with DMSO, panobinostat, JQ1 or panobinostat/JQ1 at the indicated concentrations for 48 h using a Cytotox-Glo assay. Blue or red asterisks indicate the *P*-values of panobinostat or JQ1 treatment alone compared with the combined drug treatment respectively. *p < 0.05, ** *p* < 0.01, *** *p* < 0.001, Students’ two-tailed t-test

To examine the inhibitory effect of panobinostat and JQ1 on the proliferation of GBM cells, U87, U251 and serum-free cultured U87 cells were treated with control DMSO, panobinostat, JQ1 or a combination of panobinostat and JQ1 for 24 h at the indicated concentrations, followed by staining with EdU and a flow cytometry analysis. As shown in Fig. 2a, while the treatment with panobinostat or JQ1 alone reduced the percentage of cells positively stained with EDU, the combination therapy with panobinostat and JQ1 reduced the percentage of cells positively stained with EDU more potently in each cell line. To determine whether panobinostat and JQ1 commonly induce apoptosis in GBM cells, U87, U251 and serum-free cultured U87 cells were treated with control DMSO, panobinostat, JQ1 or a combination of panobinostat and JQ1 for 48 h, followed by staining with Annexin V and a flow cytometry analysis. As shown in Fig. 2b, while the treatment with panobinostat or JQ1 alone increased the percentage of cells positively stained with Annexin V, the combination therapy with panobinostat and JQ1 increased the percentage of cells positively stained with Annexin V to 72.4% in the U87 cells, 72.9% in the U251 cells and 95.2% in the serum-free cultured U87 cells. Then, we detected caspase activity and cytotoxicity in U87, U251 and serum-free cultured U87 cells treated with DMSO, panobinostat, JQ1 or a combination of panobinostat and JQ1 for 48 h. As shown in Fig. 2c, d, compared with the panobinostat or JQ1 treatment alone, the cotreatment with panobinostat and JQ1 significantly increased caspase activity and cytotoxicity in all GBM cell lines used. These results suggest that the cotreatment with panobinostat and JQ1 markedly inhibited cell proliferation and induces apoptosis in GBM cells.

### Cotreatment with panobinostat and JQ1 commonly downregulates oncogenic gene expression in GBM

**Fig. 3:**
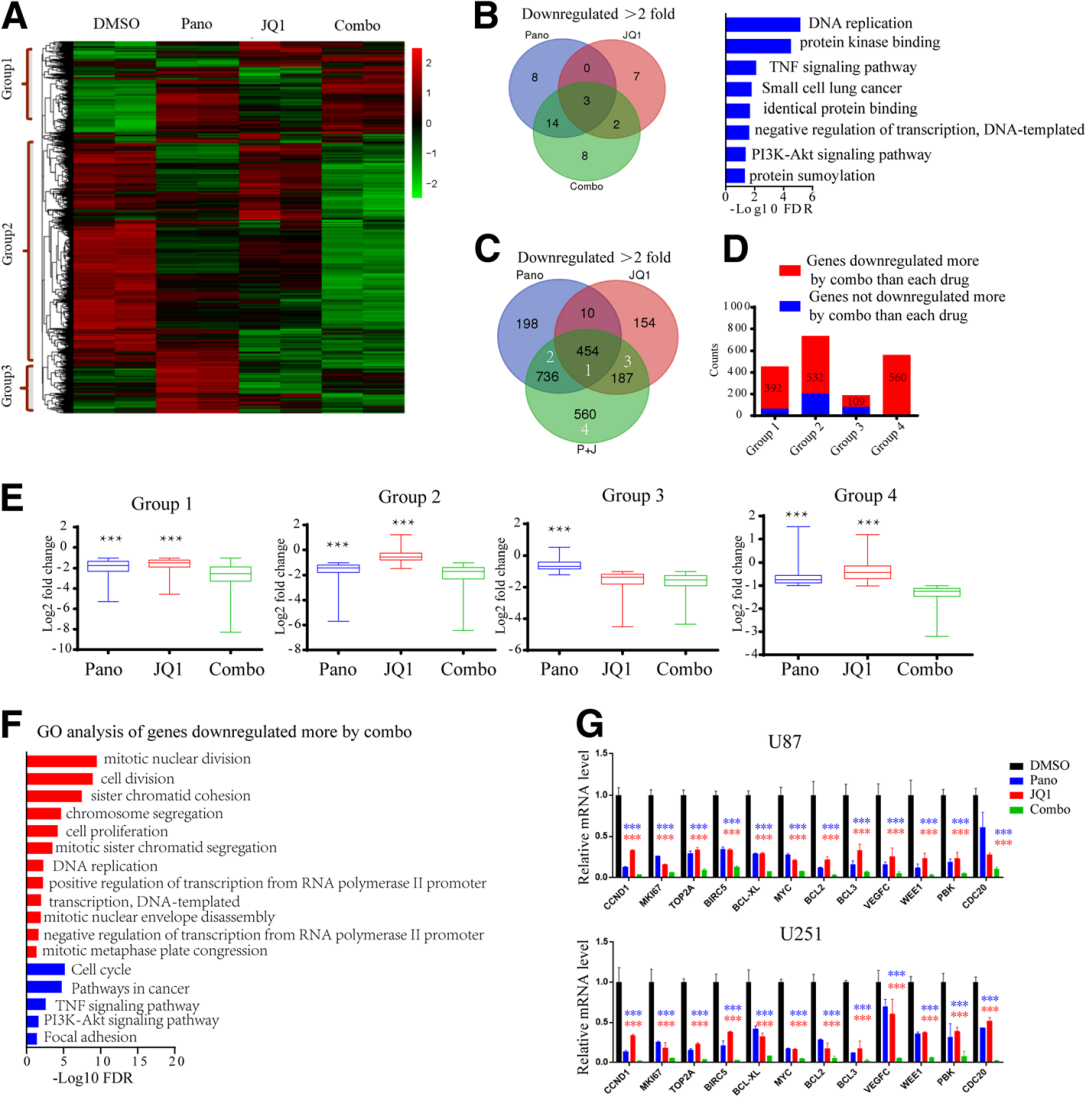
Cotreatment with panobinostat and JQ1 commonly downregulates target gene expression in GBM. **a** Heatmap of relative gene expression levels of all active transcripts in U87 cells treated with DMSO, panobinostat, JQ1 or panobinostat/JQ1 for 16 h. **b** Venn diagram showing the Gene Ontology (GO) analysis results of the transcripts significantly downregulated (log2FC ≤ − 1, FDR ≤ 0.05) by panobinostat, JQ1 or panobinostat/JQ1 treatment. In addition, the 8 gene sets downregulated by only the Pano/JQ1 combinatorial treatment are shown in the bar chart on the right. **c** Venn diagram showing the number of genes significantly downregulated (log2FC ≤ − 1, FDR ≤ 0.05) by panobinostat, JQ1 or panobinostat/JQ1 treatment. **d** Histogram showing the number of active transcripts downregulated to a greater extent by the combinatorial treatment compared with each drug treatment alone in each group shown in (**c**). **e** Box plots of log2-fold changes of genes downregulated by panobinostat, JQ1 or panobinostat/JQ1 treatment in each group shown in (**c**), (***P < 0.001 compared to the panobinostat/JQ1 treatment, Students’ two-tailed t-test). **f** GO biological processes and KEGG categories of genes downregulated to a greater extent by the combinatorial treatment compared with each drug treatment alone. **g** qPCR results showing that GBM-associated oncogenic genes related to the mitotic nuclear division, cell division and cell cycle, etc. were significantly downregulated by the combined drug treatment compared with each drug treatment alone in U87 and U251 cells. Blue or red asterisks indicate *P*-values of panobinostat or JQ1 treatment alone compared with the combined drug treatment respectively. *p < 0.05, ** *p* < 0.01, *** *p* < 0.001, Students’ two-tailed t-test

As HDAC inhibitors and bromodomain inhibitors exert anticancer effects by modulating gene expression, we studied the effect of vehicle, panobinostat alone, JQ1 alone, and combination panobinostat/JQ1 therapy on the transcriptional output of U87 cells. The RNA sequencing analysis showed a clear segregation and clustering of all groups (Fig. 3a). Importantly, among these genes, we identified three distinct clusters of DEGs associated with specific treatment conditions. As shown in Fig. 3a, the genes in group 1 were mostly upregulated in U87 cells treated with panobinostat in combination with JQ1. Group 2 contained genes with downregulated expression upon the combined panobinostat/JQ1 inhibition. The genes that were not up- or downregulated by panobinostat/JQ1 inhibition were included in group 3.

As shown in Fig. 3b, the Gene Ontology (GO) analyses of the significantly downregulated transcripts (log2FC ≤ − 1, FDR ≤ 0.05) revealed an overlap of gene functions in the panobinostat-, JQ1- or Pano/JQ1-treated U87 cells. In addition, 8 gene sets were associated with tumorigenesis in the panobinostat/JQ1 combinatorial treatment samples, such as the TNF signaling pathway, PI3K/mTOR pathway, etc. As shown in Fig. 3c, JQ1 downregulated the expression of 33.2% (464/1398) of the genes suppressed by panobinostat, and panobinostat downregulated the expression of 57.6% (464/805) of the genes suppressed by JQ1. These data suggest that JQ1 and panobinostat commonly repress target gene expression in GBM cells.

Importantly, as shown in Fig. 3d-e, 97.8% (454/464) of the genes commonly downregulated by panobinostat and JQ1 are downregulated by the combined drug treatment to a greater degree (*P*<0.001). In addition, 78.7% (736/934) of the genes only downregulated by panobinostat are downregulated by the combined drug treatment to a greater degree (*P*<0.001). In total, 54.8% of the genes only downregulated by JQ1 are downregulated by the combined drug treatment. Additionally, 560 genes were only downregulated by the combined drug treatment. GO analysis of these genes revealed that their functions are mainly oncogenesis related. Compared with both the panobinostat or JQ1 alone drug treatments, 82.2% (1593/1937) of the genes were downregulated to a greater extent by the cotreatment with panobinostat and JQ1 (Fig. 3d). The GO analysis of these genes showed that their functions were related to the mitotic nuclear division, cell division and cell cycle, etc. (Fig. 3f). The qPCR results showed that compared with the individual drug treatments, markers related to these gene functions, such as CCND1, MKI67 and TOP2A, et al., were significantly downregulated by the combined drug treatment (Fig. 3g). These genes were all reported to be oncogenic genes in GBM or glioma [24–30].

### Cotreatment with panobinostat and JQ1 results higher induction of GBM-associated tumor-suppressive genes in GBM cells

**Fig. 4:**
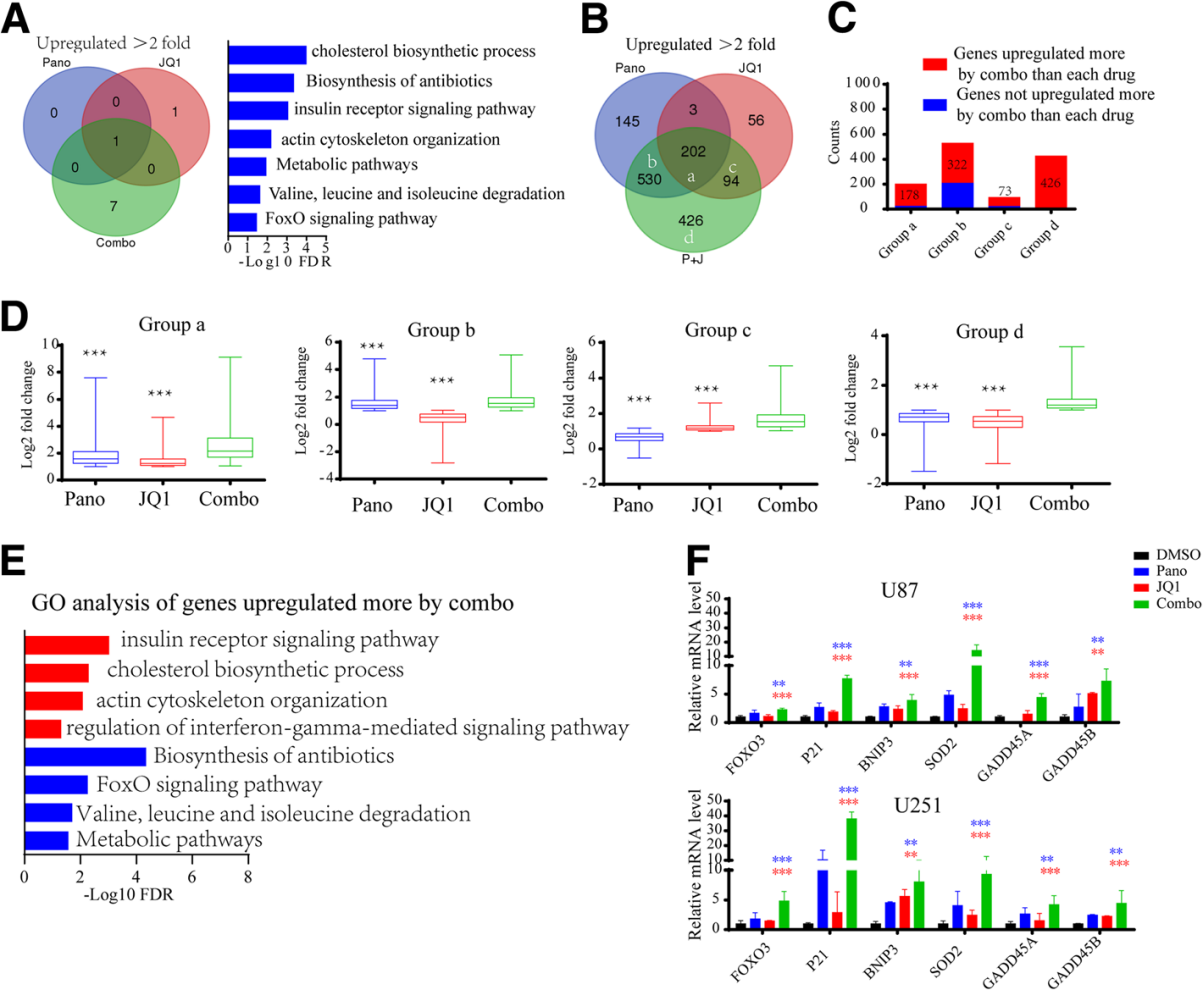
Cotreatment with panobinostat and JQ1 commonly upregulates target gene expression in GBM. **a** Venn diagram showing the Gene Ontology (GO) analysis results of the transcripts significantly upregulated (log2FC ≥ 1, FDR ≤ 0.05) by panobinostat, JQ1 or panobinostat/JQ1 treatment. In addition, 7 gene sets upregulated only by the panobinostat/JQ1 combinatorial treatment are shown in the bar chart on the right. **b** Venn diagram showing the number of genes significantly upregulated (log2FC ≥ 1, FDR ≤ 0.05) by panobinostat, JQ1 or panobinostat/JQ1 treatment. **c** Histogram showing the number of active transcripts upregulated to a greater extent by the combinatorial treatment compared with each drug treatment alone in each group shown in **b**. **d** Box plots of log2-fold changes of the genes upregulated by panobinostat, JQ1 or panobinostat/JQ1 treatment in each group shown in (**b**), (***P < 0.001 compared to the panobinostat/JQ1 treatment, Students’ two-tailed t-test). **e** GO biological processes and KEGG categories of genes upregulated to a greater extent by the combinatorial treatment compared with each drug treatment alone. **f** qPCR results showing that GBM-associated tumor-suppressive genes related to the FoxO signaling pathway and regulation of interferon-gamma-mediated signaling pathway were significantly upregulated by the combined drug treatment compared with each drug treatment alone in U87 and U251 cells. Blue or red asterisks indicate *P*-values of panobinostat or JQ1 treatment alone compared with the combined drug treatment respectively. *p < 0.05, ** *p* < 0.01, *** *p* < 0.001, Students’ two-tailed t-test

As shown in Fig. 4a, the GO analyses of the significantly upregulated transcripts (log2FC ≥ 1, FDR ≤ 0.05) showed that there is only one gene set (protein binding) shared by the panobinostat-, JQ1- and panobinostat/JQ1-treated U87 cells. However, 7 new gene sets were associated with tumor suppressors or metabolic pathways upregulated by the panobinostat/JQ1 combinatorial treatment, such as the biosynthesis of antibiotics, insulin receptor signaling pathway, and FoxO signaling pathway. As shown in Fig. 4b, JQ1 upregulated the expression of 23.3% (205/880) of the genes activated by panobinostat, and panobinostat upregulated the expression of 57.7% (205/355) of the genes activated by JQ1. These data suggest that JQ1 and panobinostat commonly activate target gene expression in GBM cells.

Importantly, as shown in Fig. 4b-d, 98.5% (202/205) of the genes commonly upregulated by panobinostat and JQ1 are upregulated by the combined drug treatment to a greater extent (*P*<0.001). In addition, 78.5% (530/675) of the genes only upregulated by panobinostat are upregulated by the combined drug treatment to a greater extent (*P*<0.001). Furthermore, 62.7% (94/150) of the genes only upregulated by JQ1 are upregulated by the combined drug treatment to a greater extent (*P*<0.001). Additionally, 426 genes were only upregulated by the combined drug treatment. GO analysis of these genes revealed that their functions are tumor-suppressor- or tumor metabolism-related. Compared with both the panobinostat or JQ1 alone drug treatments, 79.8% (999/1252) of the genes were upregulated to a greater extent by the cotreatment with panobinostat and JQ1 (Fig. 4c). The GO analysis of these genes showed that their functions were related to the insulin receptor signaling pathway, Biosynthesis of antibiotics and FoxO signaling pathway (Fig. 4e). The qPCR results showed that compared with the individual drug treatments, markers related to these gene functions, such as FOXO3, P21 and BNIP3, et at., were significantly upregulated by the combined drug treatment (Fig. 4f). These genes were all reported to be tumor suppressor genes in glioma [31–35].

### Cotreatment with panobinostat and another bromodomain inhibitor OTX015 synergistically suppresses cell growth in GBM cells

**Fig. 5:**
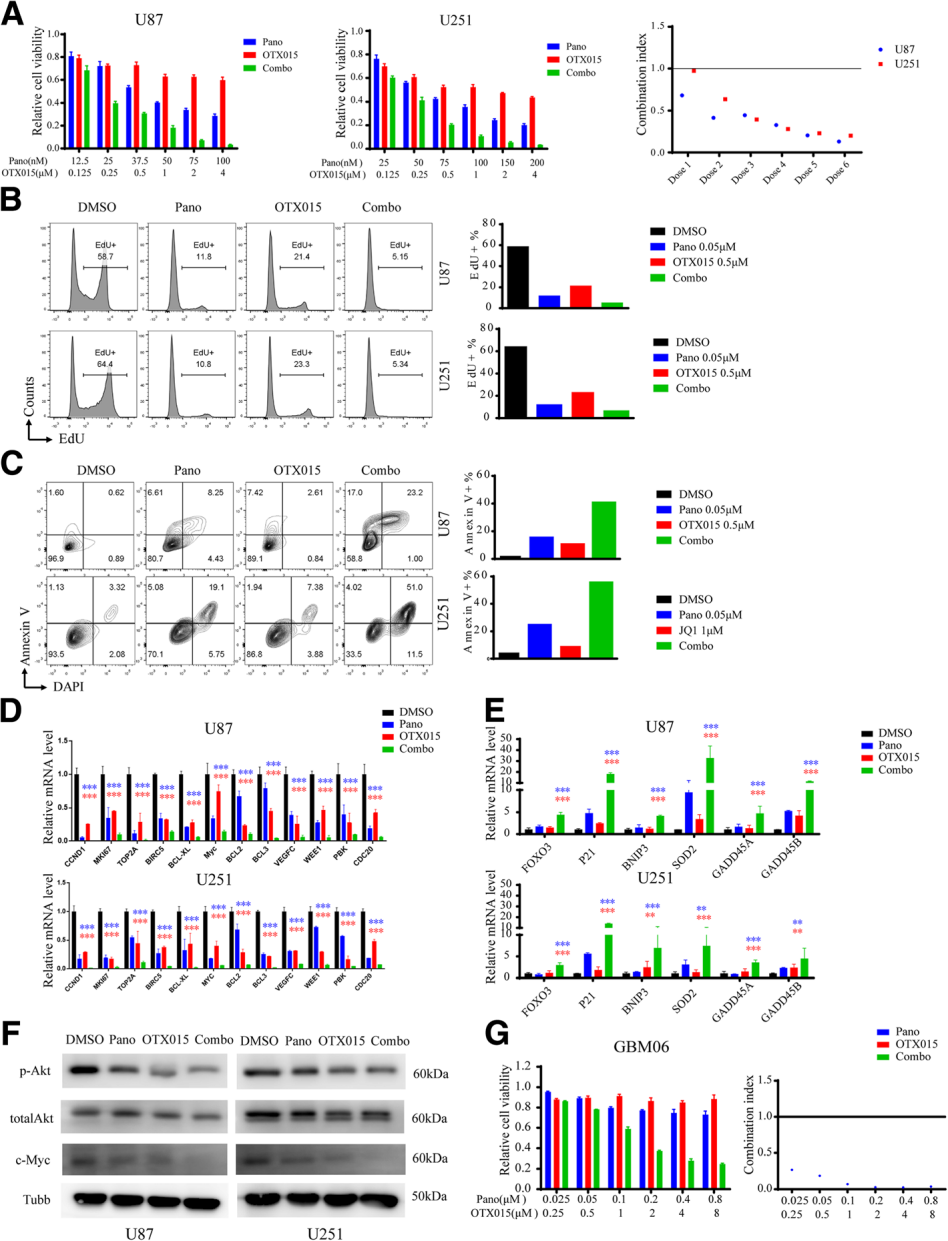
Cotreatment with the HDAC inhibitor panobinostat and the bromodomain inhibitor OTX015 synergistically suppresses cell growth in GBM cells. **a** Cotreatment with the HDAC inhibitor panobinostat and the bromodomain inhibitor OTX015 at various indicated dosages synergistically inhibits cell viability in U87 and U251 cells. The combination indices are shown on the left. **b** Cell proliferation analyses of GBM cells treated with DMSO, panobinostat, OTX015 or panobinostat/OTX015 for 24 h using an EdU incorporation FACS assay. Percentages of EdU+ cells are presented on the bar chart on the right. **c** Apoptosis analyses of GBM cells treated with DMSO, panobinostat, OTX015 or panobinostat/OTX015 for 48 h by an Annexin-V staining FACS assay. Percentages of Annexin-V+ cells are presented on the bar chart on the right. **d** qPCR results showing that GBM-associated oncogenic genes related to the mitotic nuclear division, cell division and cell cycle, etc. were significantly downregulated by the combined drug treatment compared with each drug treatment alone in U87 and U251 cells. Blue or red asterisks indicate *P*-values of panobinsotat or OTX015 treatment alone compared with the combined drug treatment respectively. *p < 0.05, ** *p* < 0.01, *** *p* < 0.001, Students’ two-tailed t-test. **e** qPCR results showing that GBM-associated tumor-suppressive genes related to the FoxO signaling pathway and regulation of interferon-gamma-mediated signaling pathway were significantly upregulated by the combined drug treatment compared with each drug treatment alone in U87 and U251 cells. Blue or red asterisks indicate *P*-values of panobinsotat or OTX015 treatment alone compared with the combined drug treatment respectively. *p < 0.05, ** *p* < 0.01, *** *p* < 0.001, Students’ two-tailed t-test. **f** Immunoblotting analyses of p-AKT, AKT and c-Myc in GBM cells treated with DMSO, panobinostat, OTX015 or panobinostat/OTX015 for 16 h. **g** Cotreatment with the HDAC inhibitor panobinostat and the bromodomain inhibitor OTX015 at various indicated dosages synergistically inhibits cell viability in primary GBM cell line. The combination indices are shown on the left

In one of our previous studies, we have shown that panobinsotat levels could achieved 200 nM in the brain following a single 20 mg/kg intraperitoneal (i.p.) dose [36]. Panobinostat also has been tested in clinical II trials of GBM patients [12]. However, JQ1 is not being tested in clinical trials due to its short half-life. Therefore, we tested the inhibitory effects of cotreatment with panobinostat with another bromodomain inhibitor OTX015 which has been proved to be effective in orthotopic glioma models and was also tested in clinical trial [37]. Berenguer-Daiz et al. has found that, in the orthotopic model, OTX015 levels achieved in tumor tissue were 995.0 ng/g (~ 2 μM), ten-times more than the levels in normal brain tissue (97.1 ng/g, ~ 200 nM) which was close to the OTX015 levels achieved in normal brain tissue (150 nM) in one of our previous studies [38]. As shown in Fig. 5a-c, cotreatment with panobinostat and OTX015 synergistically suppressed cell growth, markedly inhibited cell proliferation and induced apoptosis in GBM cells. The qPCR results showed that compared with the individual drug treatments, the oncogenic genes in glioma, such as CCND1, MKI67 and TOP2A, et al., were also significantly downregulated by the combined drug treatment (Fig. 5d), and tumor suppressor genes in glioma such as FOXO3, P21 and BNIP3, et al., were significantly upregulated by the combined drug treatment (Fig. 5e). Our Western Blot results showed that cotreatment of panobinsotat and OTX015 resulted a pronounced dephosphorylation of AKT and decreased expression level of c-Myc after 16 h of drug treatment in U87 and U251 cells (Fig. 5f). To investigate the combined inhibitory effects of panobinostat and OTX015 in primary GBM cell models, we exposed primary GBM cell line, GBM06, to increasing concentrations of panobinostat or OTX015 for 72 h. As shown in Fig. 5g, the combined treatment with panobinostat and OTX015 exerted a highly synergistic inhibition effect on primary GBM cells with a CI value well below 1.

## Discussion

Recent studies have indicated that a combined treatment with a bromodomain inhibitor and a HDAC inhibitor may be more efficacious than single drug treatments in several cancer types. Fiskus et al. reported that by inducing hyperacetylation of lysine residues on histone proteins, panobinostat could induce greater dependency on the BRD4-regulated transcription of oncoproteins in acute myelogenous leukemia (AML) such that cotreatment with panobinostat and JQ1 synergistically leads to growth inhibition and apoptosis in cultured and primary AML cells [16]. The works published by Shahbazi et al. illustrated that the bromodomain inhibitor JQ1 and panobinostat synergistically reduce N-Myc expression and induce anticancer effects in neuroblastoma [17]. In the present study, we also found that the HDAC inhibitor panobinostat and the bromodomain inhibitor JQ1 exerted synergistic anti-tumor activity against GBM cells. The cotreatment with panobinostat and JQ1 synergistically inhibited cell viability, markedly inhibited cell proliferation and induced apoptosis with elevated caspase activity and cytotoxicity in GBM cells. Importantly, we also measured the combinatory effect of HDAC inhibitor panobinostat with a clinically-available BET inhibitor OTX015 against GBM cell lines and demonstrated that they also exhibit synergistic inhibitory effects.

Bromodomain inhibitors exert biological effects by dislodging the acetylated histone readers BRD3 and BRD4 from chromatin, leading to the transcriptional repression of oncogenes [14]. In comparison, HDAC inhibitors exert biological effects by blocking the function of HDACs, leading to the transcriptional activation of tumor suppressor genes [8]. Shahbazi et al. found that bromodomain inhibitors and HDAC inhibitors commonly activate and more considerably commonly reduce target gene expression in neuroblastoma cells. Fiskus et al. reported that cotreatment with JQ1 and the HDAC inhibitor panobinostat synergistically induced apoptosis associated with a greater attenuation of oncogenes, such as c-MYC and BCL2 [16]. Shahbazi et al. also found that JQ1 and panobinostat synergistically and considerably reduced N-Myc and BCL2 expression and blocked tumor progression in neuroblastoma cells [17]. In the current study, we also found that the combination of HDAC inhibitor panobinsotat with BET inhibitor JQ1 or OTX015 results in stronger repression of GBM-associated oncogenic genes or pathways as well as higher induction of GBM-associated tumor-suppressive genes.

Based on our and other’s previous published data, both panobinostat and OTX015 could cross the blood-brain barrier at the concentrations used in our study, supporting the potential clinical application of combination therapy against GBM with these two epigenetic drugs [9, 36, 37]. In one of our previous studies, we have shown that panobinsotat levels could achieved 200 nM in the brain following a single 20 mg/kg intraperitoneal (i.p.) dose [36]. Panobinostat also has been tested in clinical II trials of GBM patients [12]. OTX015 which has been proved to be effective in orthotopic glioma models and was also tested in clinical trial [37]. Berenguer-Daiz et al. has found that, in the orthotopic model, OTX015 levels achieved in tumor tissue were 995.0 ng/g (~ 2 μM), ten-times more than the levels in normal brain tissue (97.1 ng/g, ~ 200 nM) which was close to the OTX015 levels achieved in normal brain tissue (150 nM) in one of our previous studies [38]. To further explore the potential clinical application of these two epigenetic drugs, we established one primary GBM cell lines, GBM06, from a GBM patient. We found that the combined treatment with panobinostat and OTX015 also exerted a highly synergistic inhibition effect on primary GBM cells.

## Conclusion

Our study demonstrated that HDAC inhibitor and bromodomain inhibitor had synergistical efficacy against GBM cells. HDAC inhibitor and bromodomain inhibitor commonly activate and more considerably commonly reduce target gene expression in GBM cells. The cotreatment with HDAC inhibitor and bromodomain inhibitor warrants further attention in GBM therapy.

## Abbreviations

AML: Acute myelogenous leukemia
DAPI: 4′,6-diamidino-2-phenylindole
DDR: DNA damage repair
DMSO: Dimethyl sulfoxide
FACS: Fluorescence-activated cell sorting
FDR: False discovery rate
FOXO: Forkhead box O
FPKM: Fragments per kilobase million
GBM: Glioblastoma
GO: Gene oncology
HDAC: Histone deacetylase
KEGG: Kyoto encyclopedia of genes and genomes
log2 FC: Log2-fold change
qPCR: Quantitative polymerase chain reaction
RIN: RNA Integrity number
STAR: Spliced transcripts alignment to a reference
TNF: Tumor necrosis factor
